# Information Needs for HPV Vaccination Among Different Female Population Groups in China

**DOI:** 10.1038/s41598-026-38165-8

**Published:** 2026-02-14

**Authors:** Xianglong Li, Yihan Hu, Lei Zhang, Li Du, Longmei Jin, Ping Zhou

**Affiliations:** 1 School of Public Health, National Health Commission of the People’s Republic of China Key Laboratory of Health Technology Assessment, Fudan University, Shanghai, 200032 China; 2 Shanghai Rescarch Center for Governance of Emerging Technologies in Medicine and Public Health, Shanghai, 200032 China; 3 Minhang District Maternal and Child Health Hospital, Shanghai, 201102 China; 4 Shanghai Center for Women and Children’s Health, Shanghai, 200062 China

**Keywords:** Information needs, HPV vaccine, Best-worst scaling, Diseases, Health care, Immunology

## Abstract

The current HPV vaccination rate among women is still relatively low, especially among adolescent girls aged 9–14 years. Insufficient access to HPV vaccine information is one of the important reasons, but there is a lack of research on women’s HPV vaccine information needs. What information do women truly need to support their decision to receive the HPV vaccine? The similarities and differences in the needs of different women are key issues worth studying. Clarifying this issue can provide a scientific reference for health education and help improve its effectiveness. Unlike traditional qualitative research, the purpose of this study is to quantify women’s needs for various information related to HPV vaccines, determine the priority order of information needs through quantitative methods, and explore the similarities and differences in information needs among different women with different knowledge levels and willingness to vaccinate. This study conducted a best-worst scaling experiment to compare the preference order of 7 HPV vaccines information among parents of girls aged 9–14 years, community residents, and outpatients of cervical clinic. The results of the best-worst scaling experiment were analyzed via count-based analysis and mixed logit regression analysis. Analysis shows that information needs for the safety of HPV vaccines, how to choose HPV vaccines and the duration of HPV vaccine immunization effect are generally considered important. Parents are more concerned about the safety of vaccines, and among outpatients, how to choose vaccines is the information they most want to know. Moreover, people with higher level of knowledge are relatively more concerned about the safety of vaccines and the duration of vaccine immunity, while those who are unwilling to receive vaccines have greater informational needs for vaccine safety. Women need more information about the safety of HPV vaccines, how to choose vaccines and the duration of immune effects. The information needs for HPV vaccines vary significantly among different female populations. Developing more targeted HPV vaccine health education according to these specific needs can better meet the information needs of women and potentially improve the coverage of HPV vaccines.

## Introduction

 Cervical cancer is the fourth most common cancer among women worldwide. In 2022, there were approximately 660,000 new cases of cervical cancer globally, resulting in approximately 350,000 deaths^1^. With increased awareness of cervical cancer prevention and the widespread adoption of screening, the incidence and mortality rates of cervical cancer have shown a significant decline in developed countries. In comparison, although the incidence rate of cervical cancer in China has also slowed down in recent years, it still shows an overall upward trend^2,3^. Persistent infection with the human papillomavirus (HPV) is the primary cause of cervical cancer. HPV is a common sexually transmitted virus, and nearly everyone who is sexually active will be infected at some point in their lives, usually without symptoms. Approximately 95% of cervical cancer cases are caused by prolonged HPV infection of the cervix^1^. Therefore, the World Health Organization(WHO)recommends vaccination against HPV as an effective strategy for preventing cervical cancer, with a position statement advocating for prioritizing girls aged 9 to 14 years for vaccination^4^. WHO has developed a global strategy to eliminate cervical cancer, which aims to achieve 90% of girls receiving the HPV vaccine before the age of 15 by 2030. Currently, the HPV vaccine has been introduced in over 100 countries and regions^5^.

HPV has multiple genotypes, with at least 13 high-risk HPV genotypes associated with an increased risk of cervical cancer^6^. Correspondingly, HPV vaccines also come in different valencies: bivalent vaccine (targeting HPV16 and18), quadrivalent vaccine (targeting HPV6,11,16, and 18), and nine-valent vaccine (targeting HPV 6, 11, 16, 18, 31, 33, 45, 52, and 58)^7^. All five HPV vaccines currently available in China have been shown to be effective in preventing persistent HPV infections and pre-cancerous lesions, with acceptable safety profiles. However, the HPV vaccination in China remains relatively low rate^8^. The vaccination rate among females aged 9 to 45 years is less than 0.05%, with fewer than 5% of girls aged 9 to14 years vaccinated. There are also significant variations in vaccination rates among different provinces and cities. Economically developed regions have relatively high rates, for example, the HPV vaccination rate is about 7% in Beijing and Shanghai, and 4.68% in Zhejiang Province^9^. On one hand, the HPV vaccine is not yet included in the national expanded immunization program, meaning that most parents still need to pay out of pocket for their children’s vaccinations. On the other hand, the introduction of the HPV vaccine in China has been relatively recent, so the understanding and awareness of HPV vaccine among Chinese adolescents, college students, and parents remain limited, leading to lower acceptance rates of the vaccines. Multiple studies have shown that having a good knowledge of cervical cancer and HPV vaccine and a positive attitude towards HPV vaccination, are important factors in enhancing the willingness to receive the HPV vaccine^10–13^. Therefore, there is still a need to strengthen health education for students and parents to improve their understanding of cervical cancer and HPV vaccine.

Whether HPV vaccines are self-funded or provided for free, education and access to information about HPV vaccine are the most important factors influencing willingness to vaccinate^14^. Providing information related to cervical cancer and HPV vaccine is crucial for improving health decision-making and behaviors. And in the process of health education, tailoring health information has consistently proven to be an effective strategy for improving adherence to various preventive health behaviors across diverse populations. Supporting this, health education research by Dempsey et al. demonstrated that tailored materials outperformed non-tailored materials in increasing HPV vaccination intention^15^. Therefore, although current health education interventions can improve the vaccination rate of HPV vaccines, health education tailored to the characteristics of different populations is still needed in the future^16,17^. However, studies on women’s information needs for HPV vaccines are limited. Significantly, adolescent girls are a special group. Parents’ attitudes towards the HPV vaccine, the sources of information they access, and their trust in those sources are highly correlated with adolescents’ HPV vaccination uptake^18^.

Unlike traditional qualitative studies, to understand the information needs of women for HPV vaccines, this study aimed to examine the preferences for HPV vaccine information from the perspective of parents, residents and outpatients in cervical clinics. The findings were intended to provide a scientific basis for improving health education initiatives and to assist clinicians in better understanding patient needs, ultimately promoting shared decision-making. Additionally, since women with varying levels of knowledge regarding HPV vaccines or willingness to vaccinate may have different understandings and views on HPV vaccine, this study also analyzed the similarities and differences in the information needs of different types of women based on knowledge and willingness to vaccinate. The preference analysis method adopted in this study is the Best-Worst Scaling (BWS), which was originally used to measure value preferences in the field of public policy. In recent years, BWS has become increasingly popular in various fields including health, and has unique advantages in preference measurement^19^. The best‒-worst scaling method is based on random utility theory, where respondents do not need to provide a complete ranking of all objects but only need to make discrete choices^20^.

## Methods

### Research design and participants

We conducted a cross-sectional survey in May 2024 among parents of 9–14-year-old schoolgirls, residents and outpatients aged 18–45 in a district of Shanghai, assessing their preferences for HPV vaccine information, knowledge levels regarding HPV vaccine and willingness to vaccinate. This district is located around the central urban area of Shanghai, combining both urban and suburban characteristics, providing good representativeness. There were 3 urban schools and 4 suburban schools in the district sampled. We investigated the parents of girls from grades 4 to second grade of junior high. The inclusion criteria for study participants are as follows: (1) Parents whose daughters are aged 9–14 years, and community female residents and outpatients of cervical clinic aged 18–45 years; (2) Individuals with full capacity for conduct, able to communicate normally, and capable of reading the questionnaire. The following subjects were excluded from the survey in this study: (1) those with cognitive or communication impairments who cannot understand the content of the questionnaire; (2) those with severe mental illnesses (such as schizophrenia and major depressive disorder); (3) those with other serious illnesses (such as end-stage renal failure and active cancer).

### Information needs identification

Based on a review of existing literature and consultations with two specialist clinical physicians in the field of cervical cancer^21–29^, this study identified seven key information needs regarding the HPV vaccine that women concerned about (Table 1). Based on the identified information needs, this study utilized a balanced incomplete block design, the most commonly used design to optimize the choice sets^30^, and used R 4.3.3 (R Foundation for Statistical Computing) to establish the choice sets for BWS. The respondents needed to select the information they most wanted to know and the information they least wanted to know among 4 HPV vaccine information. Prior to the survey, our research team adopted the convenient sampling method to invite 15 women aged 18–45 years to participate in the pre-survey of the questionnaire. After they filled out the questionnaire, interviews were conducted to inquire about the parts they found difficult to understand and suggestions for improving the questionnaire, in order to enhance the readability and comprehensibility of the questionnaire.

**Table 1:** Information needs in best-worst scaling experiment.

Number	Information needs
1	How to choose an HPV vaccine, such as which type to select? domestic or imported?
2	The immune effect of HPV vaccine and whether cervical cancer screening is still necessary after vaccination
3	The duration of HPV vaccine immunization effect and whether supplementary vaccination is needed
4	The safety of HPV vaccine
5	The most suitable population group and age for HPV vaccination
6	Precautions for HPV vaccination, such as whether HPV testing should be done in advance
7	The routes to obtain vaccine and prices of HPV vaccination

### Questionnaire design

The survey questionnaire consisted of four sections:

Demographic characteristics of the respondents: This section consisted of questions about respondents’ age, marital status, education level, primary occupation, annual household income, family history related to HPV and cervical cancer.

Preferences for HPV vaccine information based on BWS: Respondents need to select the information they most wanted to know and the information they least wanted to know. Each respondent completed 7 choice tasks, each choice task consisting of 4 HPV vaccine information.

Knowledge about Cervical Cancer and HPV vaccine: This part assessed respondents’ knowledge literacy by referring to knowledge scales for cervical cancer, HPV, HPV vaccines, and cervical cancer screening^31–33^. Two clinical experts in cervical cancer assisted in localizing and improving this scale. The revised questionnaire included 11 questions on cervical cancer (e.g., having multiple sexual partners increases cancer risk), 18 questions on HPV (e.g., HPV infections have obvious symptoms), 9 questions on HPV vaccine (e.g., only one dose of HPV vaccine is needed), and 11 questions on cervical cancer screening (e.g., screening generally starts at age 25), totaling 49 knowledge questions. The Cronbach’s α coefficients for the four sections were 0.948, 0.961, 0.885, and 0.95, respectively, with an overall Cronbach’s α coefficient of 0.965.

Willingness to vaccinate with HPV Vaccine: This part assessed parents’ willingness to vaccinate their daughters including whether they had made an appointment for their daughter’s HPV vaccination. Residents and outpatients answered whether they were willing to receive the HPV vaccine for themselves.

### Ethical approval and data collection

The study was ethically reviewed and approved by the Minhang District Maternal and Child Health Hospital Research Ethics Committee (2024HS-02). All methods in this study were conducted in accordance with the Declaration of Helsinki. We confirm that we have obtained informed consent from all participants. This study employed an anonymous survey method, and the survey content did not involve the identifiable information of the participants. Respondents were free to choose whether to complete the questionnaire after reading the research instructions.

An online survey questionnaire was designed on the Questionnaire Star platform. Prior to distribution, the questionnaire underwent expert consultation and a pilot study to ensure its quality. Professionals from the maternal and child health hospital provided unified training to the staff responsible for student affairs in the Social Affairs Office and family doctors. The questionnaire was then distributed in the form of QR codes to the parent groups corresponding to the target grades in the schools. Residents were contacted by their family doctors to fill out questionnaires, while outpatients were randomly selected by investigators at the cervical clinic for investigation.

### Sample size

Currently, there is no formal guidance on the optimal sample size for BWS. Louviere et al. indicated in their work on BWS that although the minimum sample size for count-based BWS analyses such as BW Scores has not been established, the modelling approaches such as conditional logit estimation in bws-1 allow for a reference to the guidelines for sample size in discrete choice experiments (DCE)^34^. A commonly method used for estimating DCE samples is the thumb rule: *N* ≥ 500c/(t*a), where 500 is a fixed quantity, c is the maximum number of levels for any attribute, t is the number of choice sets in the questionnaire, and a is the number of options in each set^35^. Thus, the minimum sample size calculated for this study was18 participants. This is consistent with that more than 20 respondents per choice set could estimate reliable models^35^. From 2017 to 2021, the average sample size of BWS studies in the health field was 472, including BWS-2 and BWS-3 studies^36^. In this study, the sample sizes of parents, residents, and outpatients could provide reasonable precision in our estimates.

### Statistical analysis

Firstly, analysis focused on counting the frequency of each information need was selected as the best or worst. The calculation of BW scores was as follows: the number of times an item was chosen as the most wanted information minus the number of times it was chosen as the least wanted information. A positive BW score indicates that the information was primarily selected as most needed, while a negative score indicates the opposite. The BW scores were also standardized to compare the relative importance of different information needs.

$$BW{\text{ }}score{\text{ }} = Count_{{best}} - Count_{{worst}}$$

$$S\tan dard{\text{ }}score{\text{ }} = \frac{{Count_{{best}} - Count_{{worst}} }}{{r~ \times ~n}}$$

Secondly, this study employed mixed logit regression to analyze parents’ HPV vaccine information needs. The mixed logit model operated under the assumption that respondents are assumed to first select the most concerning option, followed by the least concerning option^37^.

Finally, subgroup analyses were conducted based on knowledge levels and willingness to get vaccinated to observe the heterogeneous preferences of different population for HPV vaccine information. Each correct answer to the question of knowledge earns 1 point, while incorrect or uncertain answers do not earn any points. Due to the non-normal distribution of knowledge scores, this study considered participants with knowledge scores exceeding or equal to 60% of the full score to have good knowledge. Data were analyzed using R 4.3.3 and Stata 18.0.

## Results

### Characteristics of surveyed parents

A total of 1,331 parent questionnaires, 844 resident questionnaires, and 484 outpatient questionnaires were collected. After excluding questionnaires with invalid response times (shorter than 4 min or longer than 20 min), the final analysis retained 1,195 valid parent questionnaires, 525 resident questionnaires, and 438 outpatient questionnaires.

The parents of this study were mainly females (Male:8.45% vs. Female:91.55%) (Table 2), with the majority of parents aged 35–45 years old (82.77%). About 83.68% of parents expressed willingness to vaccinate their daughters against HPV, but only 9.96% of parents stated that they had vaccinated their daughters or planned to vaccinate their daughters. The surveyed residents and outpatients were all female, with a higher level of education, and overall knowledge related to HPV vaccines were slightly better than parents. About 79.43% of residents and 84.93% of outpatients expressed willingness to receive the HPV vaccine. More than 40% of outpatients explicitly stated a family history of HPV infection.

**Table 2:** Characteristics of surveyed parents, residents and outpatients.

Characteristics		Parents		Residents		Outpatients	
		Number	Percentage	Number	Percentage	Number	Percentage
Gender							
Male		101	8.45%	0	0.00%	0	0.00%
Female		1094	91.55%	525	100.00%	438	100.00%
Total		1195		525		438	
Age							
<=35		119	9.96%	229	43.62%	215	49.09%
36–40		413	34.56%	103	19.62%	86	19.63%
41–45		571	47.78%	193	36.76%	137	31.28%
> 45		92	7.70%	0	0.00%	0	0.00%
Total		1195		525		438	
Nationality							
Han nationality		1146	95.90%	504	96.00%	430	98.17%
Other		49	4.10%	21	4.00%	8	1.83%
Total		1195		525		438	
Marital status							
Married		995	83.26%	440	83.81%	316	72.15%
Other		200	16.74%	85	16.19%	122	27.85%
Total		1195		525		438	
Education							
Junior high school or below		230	19.25%	31	5.90%	61	13.93%
High school or vocational school		151	12.64%	55	10.48%	85	19.41%
College degree		231	19.33%	114	21.71%	102	23.29%
Bachelor degree or above		583	48.79%	325	61.90%	190	43.38%
Total		1195		525		438	
Occupation							
Agriculture, industry or traditional service industry		329	27.53%	125	23.81%	117	26.71%
Public institution		190	15.90%	251	47.81%	73	16.67%
Employees of pharmaceutical, financial, Internet and high-tech enterprises		195	16.32%	31	5.90%	66	15.07%
Other		481	40.25%	118	22.48%	182	41.55%
Total		1195		525		438	
Annual household income							
< 100 thousand yuan		295	24.69%	136	25.90%	166	37.90%
100–300 thousand yuan		504	42.18%	315	60.00%	204	46.58%
310–500 thousand yuan		202	16.90%	54	10.29%	46	10.50%
> 500 thousand yuan		194	16.23%	20	3.81%	22	5.02%
Total		1195		525		438	
Family history of HPV infection							
Yes		55	4.60%	51	9.71%	176	40.18%
No		1077	90.13%	401	76.38%	198	45.21%
Not sure		63	5.27%	73	13.90%	64	14.61%
Total		1195		525		438	
Family history of cervical cancer							
Yes		8	0.67%	9	1.71%	13	2.97%
No		1171	97.99%	493	93.90%	379	86.53%
Not sure		16	1.34%	23	4.38%	46	10.50%
Total		1195		525		438	
Knowledge level							
Higher level of knowledge		416	34.81%	257	48.95%	218	49.77%
Lower level of knowledge		779	65.19%	268	51.05%	220	50.23%
Total		1195		525		438	
Willingness to get vaccinated							
Willing		1000	83.68%	417	79.43%	372	84.93%
Unwilling		195	16.32%	108	20.57%	66	15.07%
Total		1195		525		438	

### BWS estimates by count-based analysis

Among the three groups – parents, community residents, and outpatients – the BW scores for “The safety of HPV vaccines”, “How to choose an HPV vaccine, such as which type to select? domestic or imported?”, and “The duration of HPV vaccine immunization effect and whether supplementary vaccination is needed” were all positive (Table 3). This indicates that this information was more frequently selected as the most wanted information. Specifically, safety was the top priority for parents and residents, while outpatients were most interested in information on how to choose a vaccine.

In contrast, the BW scores for “The most suitable population group and age for HPV vaccination”, “Precautions for HPV vaccination, such as whether HPV testing should be done in advance”, and “The routes and prices of HPV vaccination” were all negative. This indicates that this information was more frequently selected as the least wanted information. Vaccination routes and prices were consistently among the least desired information items.

Overall, the information preference rankings were relatively consistent between community residents and outpatients. However, since parents were making decisions about vaccination for their daughters, their information preference order differed. Parents showed relatively greater interest in knowing about the duration of immunity and information regarding the most suitable target population and age.

**Table 3:** BWS estimates by count-based analysis.

Information needs	Parents			Residents			Outpatients		
	BW	Std.BW	Rank	BW	Std.BW	Rank	BW	Std.BW	Rank
The safety	1850	0.387	1	608	0.290	1	442	0.252	2
How to choose a vaccine	1254	0.262	2	464	0.221	2	511	0.292	1
The duration of immunization effect	450	0.094	3	185	0.088	4	14	0.008	4
The most suitable population and age	-654	-0.137	4	-594	-0.283	6	-425	-0.243	6
The immune effect	-670	-0.140	5	274	0.130	3	211	0.120	3
Precautions	-980	-0.205	6	-200	-0.095	5	-253	-0.144	5
The routes and prices	-1250	-0.262	7	-737	-0.351	7	-500	-0.285	7

### BWS estimates by modelling approaches

**Fig. 1:**
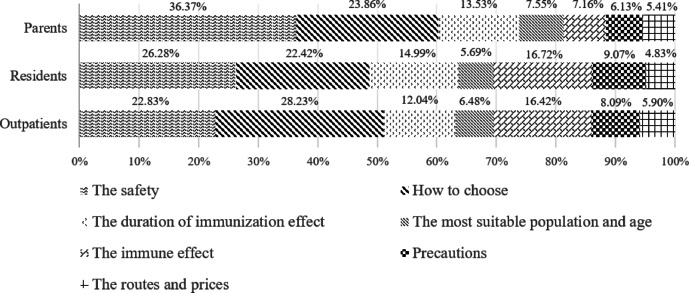
Relative importance of information for three different population groups.

The information preference order derived from the mixed logit model was consistent with the results of the count analysis. The correlation coefficients and relative importance scores for each information item are presented in Table 4; Fig. 1.

Although vaccine safety was ranked as the most preferred information item by both parents and community residents, the magnitude of their preference differed. Among parents, safety information accounted for a substantially higher preference share (36.37%) compared to residents (26.28%) and outpatients (22.83%). Conversely, among outpatients, information on how to choose a vaccine had the highest preference share (28.23% vs. 22.42% for residents and 23.86% for parents).Furthermore, community residents and outpatients demonstrated a relatively higher level of interest in information regarding vaccine immune effect (16.72% and 16.42%, respectively) compared to parents (7.16%) (Table 4.

**Table 4:** BWS estimates by modelling approaches.

Information needs	Parents			Residents			Outpatients		
	β	SE	Importance	β	SE	Importance	β	SE	Importance
The safety	1.906^***^	0.064	36.37%	1.695^***^	0.082	26.28%	1.352^***^	0.077	22.83%
How to choose a vaccine	1.485^***^	0.061	23.86%	1.536^***^	0.087	22.42%	1.565^***^	0.096	28.23%
The duration of immunization effect	0.918^***^	0.046	13.53%	1.133^***^	0.080	14.99%	0.712^***^	0.078	12.04%
The most suitable population and age	0.334^***^	0.044	7.55%	0.164^***^	0.052	5.69%	0.093	0.060	6.48%
The immune effect	0.281^***^	0.044	7.16%	1.243^***^	0.071	16.72%	1.023^***^	0.075	16.42%
Precautions	0.126^***^	0.038	6.13%	0.630^***^	0.055	9.07%	0.316^***^	0.066	8.09%
The routes and prices	ref	-	5.41%	ref	-	4.83%	ref	-	5.90%

β, coefficients; SE, standard error.

***p < 0.01,**p < 0.05,*p < 0.1.

### Heterogeneity of information needs

Heterogeneity analysis across different knowledge levels and willingness to vaccinate was conducted within the mixed logit model framework by introducing interaction terms (Tables 5 and 6). The results revealed that among parents and community residents, the coefficients for the interaction terms between the binary knowledge levels variable and the information attributes were consistently positive, indicating that parents and residents with higher knowledge level expressed a stronger desire to learn about HPV vaccine-related information. Conversely, among outpatients, no significant difference was observed between those with higher and lower knowledge levels; outpatients with lower knowledge level demonstrated a desire to learn about HPV vaccine information similar to those with higher knowledge levels. Analysis using interaction terms between the binary vaccination intention variable and information attributes showed that for parents, the statistically significant interaction coefficients were consistently negative, suggesting that parents unwilling to vaccinate their daughters showed a relatively stronger desire to learn about HPV vaccine information. In contrast, for community residents and outpatients, the statistically significant interaction coefficients were positive, indicating that residents and outpatients willing to get vaccinated expressed a stronger desire to learn about the vaccine information.

**Fig. 2:**
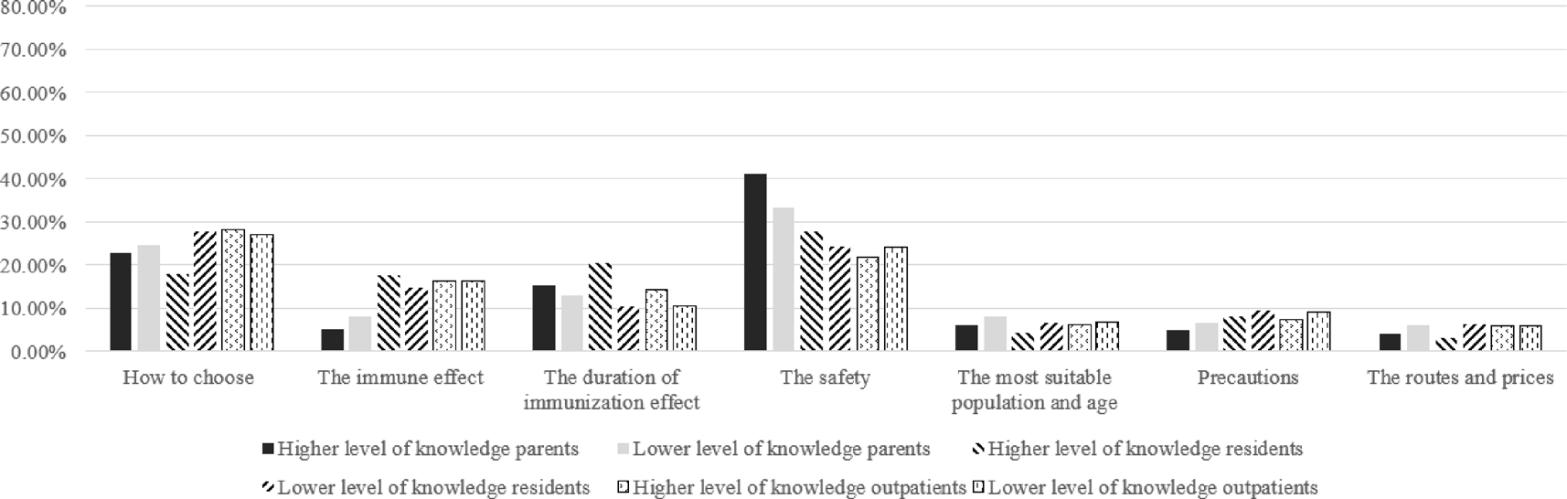
Relative importance of information for three different population groups with different knowledge levels.

**Fig. 3:**
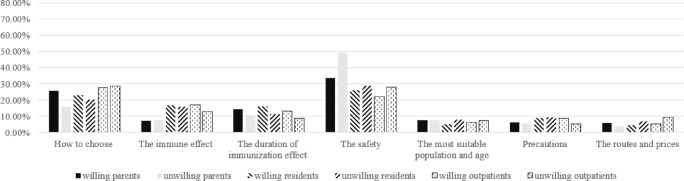
Relative importance of information for three different population groups with different willingness.

**Fig. 4:**
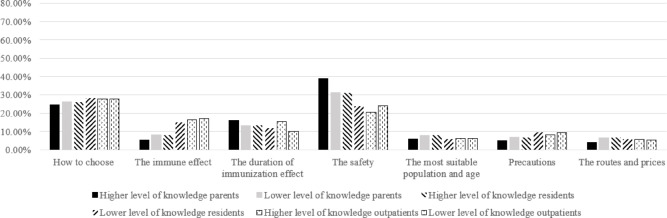
Relative importance of information for willing population groups with different knowledge levels.

**Fig. 5:**
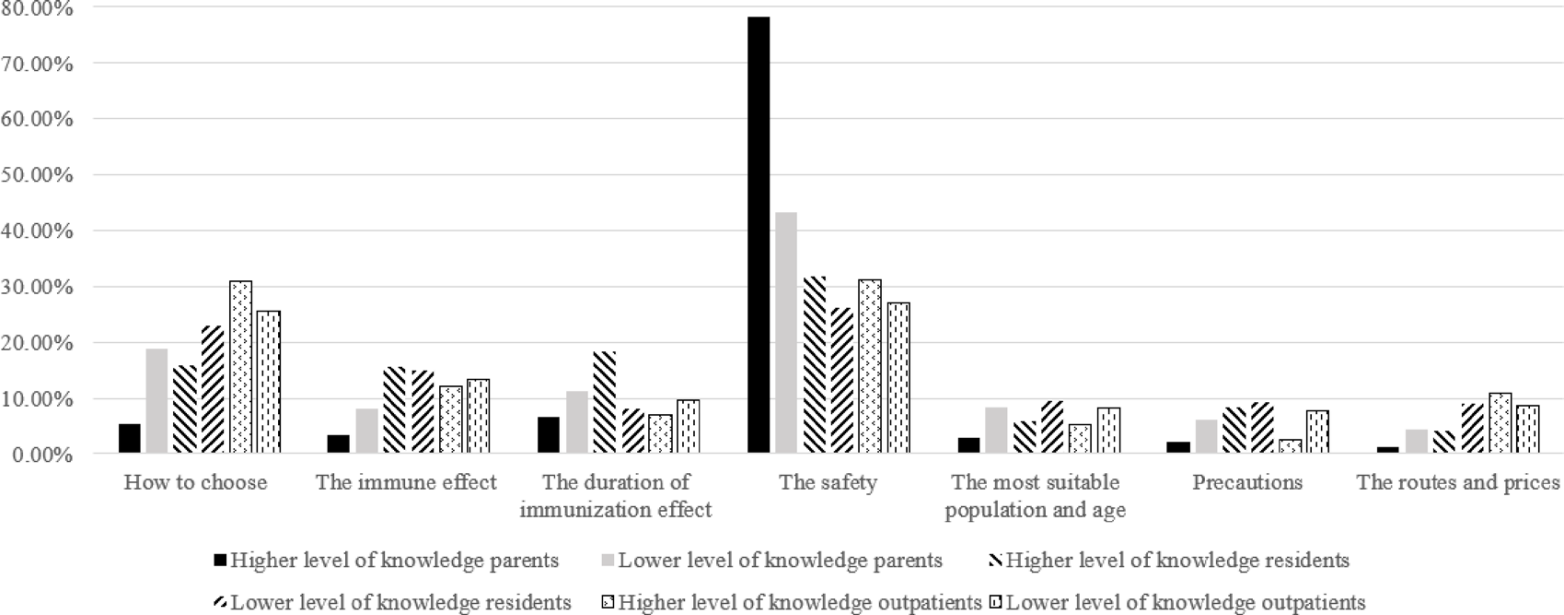
Relative importance of information for unwilling population groups with different knowledge levels.

 To examine the similarities and differences in the relative importance of information across population types, subgroup analyses were also conducted based on knowledge levels and willingness to vaccinate (Figs. 2, 3, 4 and 5). Knowledge levels subgroup analysis showed that individuals with higher knowledge level placed relatively greater importance on information regarding vaccine safety and the duration of immunization effect. Individuals with lower knowledge level placed relatively greater importance on information about how to choose a vaccine, the suitable target population and age, precautions before vaccination, cost, and vaccination route. Notably, how to choose a vaccine was ranked as the most important information among residents with lower knowledge levels and among all outpatients. Willingness to vaccinate stratification revealed that individuals unwilling to vaccinate assigned relatively greater importance to information about vaccine safety, while individuals willing to vaccinate assigned relatively greater importance to information about the duration of vaccine effectiveness and how to choose a vaccine.

**Table 5:** Heterogeneity analysis of information preference among people with different knowledge levels.（Dear Editor, from the order of the article, Tables 5 and 6 should be placed before Figures 2-5）(Important: please refer to table 4 and add the last row to table 5: the routes and prices, which is the reference level)

Response	Parents		Residents		Outpatients	
	β	SE	β	SE	β	SE
Knowledge x How to choose a vaccine	0.182	0.126	0.144	0.170	-0.001	0.185
Knowledge x The immune effect	-0.004	0.094	0.760^***^	0.139	-0.058	0.147
Knowledge x The duration of immunization effect	0.486^***^	0.097	1.266^***^	0.154	0.293^*^	0.154
Knowledge x The safety	0.391^***^	0.129	0.677^***^	0.157	-0.179	0.150
Knowledge x The most suitable population and age	0.138	0.094	0.243^**^	0.105	-0.098	0.120
Knowledge x Precautions	0.144^*^	0.082	0.471^***^	0.109	-0.233^*^	0.131
How to choose	1.446^***^	0.075	1.487^***^	0.120	1.568^***^	0.133
The immune effect	0.292^***^	0.055	0.877^***^	0.095	1.048^***^	0.105
The duration of immunization effect	0.770^***^	0.057	0.526^***^	0.105	0.563^***^	0.108
The safety	1.780^***^	0.077	1.378^***^	0.109	1.449^***^	0.108
The most suitable population and age	0.268^***^	0.055	0.045	0.072	0.148^*^	0.085
Precautions	0.072	0.048	0.405^***^	0.075	0.447^***^	0.093

β, coefficients; SE, standard error.

***p < 0.01,**p < 0.05,*p < 0.1.

Higher level of knowledge: knowledge scores exceeding or equal to 60% of the full score.

**Table 6:** Heterogeneity analysis of information preference among people with different willingness.(Important: please refer to table 4 and add the last row to table 5: the routes and prices, which is the reference level)

Response	Parents		Residents		Outpatients	
	β	SE	β	SE	β	SE
Willingness x How to choose a vaccine	0.035	0.161	0.501^**^	0.206	0.554^**^	0.259
Willingness x The immune effect	-0.527^***^	0.121	0.481^***^	0.169	0.799^***^	0.204
Willingness x The duration of immunization effect	-0.175	0.126	0.747^***^	0.192	0.959^***^	0.214
Willingness x The safety	-0.751^***^	0.167	0.288	0.192	0.286	0.210
Willingness x The most suitable population and age	-0.462^***^	0.122	-0.002	0.127	0.435^***^	0.169
Willingness x Precautions	-0.333^***^	0.105	0.422^***^	0.132	1.036^***^	0.181
How to choose	1.477^***^	0.148	1.159^***^	0.185	1.098^***^	0.240
The immune effect	0.730^***^	0.110	0.860^***^	0.150	0.340^*^	0.187
The duration of immunization effect	1.082^***^	0.115	0.538^***^	0.170	-0.102	0.197
The safety	2.539^***^	0.156	1.467^***^	0.172	1.120^***^	0.194
The most suitable population and age	0.703^***^	0.112	0.161	0.113	-0.271^*^	0.156
Precautions	0.399^***^	0.096	0.295^**^	0.117	-0.548^***^	0.167

β, coefficients; SE, standard error.

***p < 0.01,**p < 0.05,*p < 0.1.

## Discussion

 Health education is one of effective strategies for healthcare professionals to improve HPV vaccination rates^38,39^. There is an association between vaccine uptake and whether the information provided was satisfactory or thought helpful^40^. However, relevant research indicates that current health education still primarily focuses on the long-term benefits of the HPV vaccine^41^. Indeed, as Gilkey et al. found, preventing cancer is the most important reason for getting vaccinated against HPV^42^. But this does not mean that the immune effect of the vaccine is the core of the information and health education that women need. Especially among the special group of parents, in recent years, the reasons why parents hesitate to receive HPV vaccines are changing. The proportion of parents who consider “safety or side effects” as a reason for vaccine hesitancy is significantly increasing, while the proportion of parents who consider their children’s sexual inactivity or vaccine non recommendation by healthcare providers as a reason for hesitancy is decreasing year by year^43^. This is consistent with the results of our study, this study employed the Best-Worst Scaling method to quantify the information needs for HPV vaccine among parents, residents and outpatients, and indirectly revealed the reasons behind their hesitance to get vaccinated against HPV. Parents, residents and outpatients may not be unaware of the long-term benefits of the HPV vaccine but rather lack comprehensive understanding of the safety of the HPV vaccine, how to choose an HPV vaccine (such as which type to select? ), and the duration of HPV vaccine immunization effect. For parents, the most suitable age for vaccination is also a very important piece of information. These factors could be more critical reasons for women’s hesitance regarding vaccination. Therefore, health education focusing on the safety of HPV vaccine, how to choose them, long-term benefits, and the best age for vaccination (9–14 years old) may lead to more effective outcomes.

This study investigated information needs regarding the HPV vaccine among three distinct groups: parents of adolescent girls, community residents, and outpatients. The findings demonstrate significant differences in information needs across these populations. Consequently, health education initiatives should be tailored to the specific characteristics of the target audience to provide more relevant information. For instance, parents exhibited a pronounced preference for information on vaccine safety. This focus aligns with their unique role as decision-makers for their children^44^. As supported by VanWormer et al., when considering vaccination for their children rather than themselves, influenced by the child’s age and anticipated time to sexual debut, parents tend to underestimate their child’s risk of contracting sexually transmitted infections or cervical cancer and overestimate the risk of adverse events associated with the HPV vaccine^45^, leading to heightened caution regarding safety aspects. Conversely, outpatients demonstrated the lowest level of preference for safety information. For this group, information on “how to choose a vaccine” emerged as their foremost concern. This prioritization is likely linked to their immediate decision-making context: facing an existing HPV infection, concerns about disease progression may overshadow anxieties regarding vaccine safety. A similar pattern was observed in a study on willingness to receive therapeutic HIV vaccination, where safety concerns constituted the primary reason for vaccine hesitancy (37%). Notably, patients who had acquired HIV through sexual transmission or had been living with HIV for over five years exhibited significantly higher willingness to receive the therapeutic HIV vaccine^46^.

 Since women with varying levels of knowledge or different willingness to vaccinate may have different views on the HPV vaccine, this study conducted heterogeneity analyses and subgroup analyses among parents, residents and outpatients with varying knowledge and different willingness to vaccinate. HPV vaccine literacy serves as a significant predictor of HPV vaccine series completion^47^. Our analysis of knowledge heterogeneity revealed that parents and community residents with higher knowledge levels exhibited a stronger desire for information about the HPV vaccine. This suggests that a foundational understanding of cervical cancer, susceptibility to HPV, the severity of HPV-related diseases, and the benefits of vaccination may stimulate greater interest in seeking further information to achieve a more comprehensive understanding. This is consistent with the findings of Patil et al., who found that people with low health literacy are more likely to perceive the response to public health crises as an overreaction and often have overconfidence in evaluating health information^48^. Conversely, no significant difference in information preferences was observed among outpatients with varying knowledge levels. This may be attributable to their overall elevated risk perception, which likely drives a consistently high preference for vaccine-related information across this group. Regarding heterogeneity in willingness to vaccinate, compared to residents or outpatients unwilling to vaccinate, those willing to vaccinate demonstrated a relatively stronger preference for vaccine information. Interestingly, among parents, those unwilling to vaccinate their children showed a relatively stronger desire for vaccine information compared to those willing. This further underscores the unique position of parents. The responsibility of making vaccination decisions for their children heightens parental risk aversion^49^, leading to more cautious decision-making. This observation aligns with the findings synthesized by Dubé E et al., indicating that parents who decline vaccination for their children may exhibit heightened interest in health-related information^50^.

 We further conducted subgroup analyses based on knowledge level and willingness to vaccinate to examine the relative importance of different information types across population groups. Overall, individuals with higher level of HPV vaccine knowledge showed more concentrated preferences, assigning higher relative importance to vaccine safety and the duration of vaccine protection. This may relate to their existing basic understanding of HPV. Notably, excluding safety-focused parents, among lower-knowledge residents and all outpatients, information on “how to choose a vaccine” had the highest relative importance. This suggests these groups may face greater difficulty in selecting HPV vaccines. Providing targeted guidance could help convert willingness to vaccinate into actual uptake, thereby improving vaccination rates. Across willingness-based subgroups, vaccine safety held significantly higher relative importance among those unwilling to vaccinate. This indicates safety concerns may be a primary reason for their reluctance, aligning with Zhang et al.‘s review and Rositch et al.‘s findings on HPV vaccine hesitancy^51,52^.

 This study does have some limitations. First, due to the methodological requirements of BWS and considerations regarding survey feasibility, this study selected 7 key information needs that women generally care about and consider important based on literature reviews, key informant interviews, and expert consultations. Other potential information needs may not have been included in this study. Secondly, although the sample in this study took into account the differences between urban and suburban areas, it hasn’t been able to select samples comprehensively and randomly, resulting in certain limitations in sample representativeness. Furthermore, Shanghai has a relatively high overall education level and socio-economic conditions, which may affect the universality of the research results. Future research should sample on a national scale to further investigate the stability of the findings. Lastly, due to a relatively low proportion of parents in the sample explicitly stating their unwillingness to vaccinate their daughters, the sample size for subgroup analysis of the population unwilling to receive vaccines in this study is relatively small.

## Conclusions

Despite these limitations, this study provided valid evidence for analyzing HPV vaccine information needs from the perspectives of parents, community residents, and outpatients. While cancer prevention remains the primary motivation for HPV vaccination, this does not imply that the long-term benefits should be the central focus of health education. Our findings demonstrate that female populations have a greater need for information concerning: the safety HPV vaccine, how to choose a vaccine, the duration of protection, and the optimal age for vaccination. Furthermore, information needs regarding the HPV vaccine vary across different population groups. Parents exhibit particularly high concern for vaccine safety, especially those with higher knowledge levels or who are hesitant about vaccination. Conversely, residents with worse knowledge and outpatients identified information on vaccine selection as their most critical need. Based on these findings, developing more targeted HPV vaccine health education according to these specific needs can better meet the information needs of women and potentially improve the coverage of HPV vaccines.

## Data Availability

The datasets generated or analyzed during this study are available from the corresponding author on reasonable request.
